# Cognitive determinants of weight control by dietary patterns among postmenopausal women with osteoporosis: An application of theory of planned behavior

**DOI:** 10.34172/hpp.2021.57

**Published:** 2021-12-19

**Authors:** Hossein Hajizadeh, Pouria Sefidmooye Azar, Haidar Nadrian, Farhang Soltani Bejestani, Sousan Kolahi, Kritika Gupta

**Affiliations:** ^1^Nutrition Research Center, Department of Nutrition, Faculty of Nutrition and Food Sciences, Tabriz University of Medical Sciences, Tabriz, Iran; ^2^Department of Nutrition, School of Applied Sciences, The University of Mississippi, Oxford, MS, USA; ^3^Social Determinanta of Health Research Center, Tabriz University of Medical Sciences, Tabriz, Iran; ^4^Department of Rheumatology, Faculty of Medicine, Gonabad University of Medical Sciences, Gonabad, Iran; ^5^Connective Tissue Diseases Research Center, Tabriz University of Medical Sciences, Tabriz, Iran; ^6^Department of Nutrition and Hospitality Management, School of Applied Sciences, The University of Mississippi, Oxford, MS, USA

**Keywords:** Body weight change, Osteoporosis, Dietary pattern, Postmenopausal period, women

## Abstract

**Background:** In this study, we aimed to assess the cognitive determinants of weight control behaviors by dietary patterns among postmenopausal women with osteoporosis.

**Methods:** This cross-sectional study, based on the theory of planned behavior (TPB) was conducted from July to December 2017 among 240 postmenopausal women with osteoporosis in Tabriz, Iran. A validated and reliable TPB-based instrument, namely Weight-CuRB, and the food frequency questionnaire (FFQ) were used.

**Results:** The results of exploratory factor analysis (EFA) indicated three dietary patterns (total variance explained=24.44%); healthy (n=71), mixed (n=78), and western (n=91). In addition, food items consumed by participants were classified into twenty-two food groups for dietary pattern analysis. In the healthy and western dietary patterns, attitude (β: 0.140, *P* <0.001) and subjective norms (SNs) (β: 0.498, *P* <0.01) were the only predictors of weight control behavior, respectively. In the women with healthy and western patterns, the TPB-based variables altogether explained 11% and 16% of variations in the behavior, respectively. Among all patients, the TPB-based variables explained 12.2% of variations in weight control behavior, within which SNs were the only significant predictor of the behavior (β=-0.199, *P* <0.01).

**Conclusion:** Our findings highlighted the remarkable role of dietary patterns in the associations between weight control and its cognitive determinants. Dietary patterns should be considered while designing weight control educational interventions among women with osteoporosis. In such interventions, promoting SNs and perceived behavioral control (PBC) should be considered as the core strategies to promote the behaviors among the patients who follow an unhealthy diet.

## Introduction

Reduced level of estrogen in postmenopausal women is associated with weight gain, abdominal obesity, insulin resistance, and osteoporosis.^1,2^ Given the role of estrogen in bone metabolism, lower levels of estrogen increase the risk of bone fracture, movement disorders, and reduced bone mineral density (BMD) in obese postmenopausal women with osteoporosis.^3^ In addition to reduced levels of estrogen, the factors like nutrition, physical activity, and body mass index (BMI) are among the factors that influence BMD.^4,5^ Individuals with BMI either above 30 kg/m^2^ or below 18.5 kg/m^2^ are more prone to bone fractures,^6,7^ considering that a majority of fractures are often the consequence of a reduction in the protective effects of estradiol on the bone.^8,9^

Overweight and obesity are also reported as risk factors for vertebral fracture among postmenopausal osteoporotic women.^7^ Patients with BMI < 18.5 kg/m^2^ are vulnerable to hip fractures as compared to those with normal BMI (18.5-25 kg/m^2^).^10^ Nevertheless, Armstrong, et al reported that the relative risk for bone fracture was 1.71 in overweight patients (25.0-29.9 kg/m^2^) and 2.55 in patients with BMI between (20.0-24.9 kg/m^2^).^11^

Eating habits and behavioral patterns including physical activity have a major role in the prevention of bone fractures.^12^ Nutrition not only plays an important role in maintaining desirable weight but can also elevate BMD in patients with osteoporosis.^13^ Numerous studies have shown associations of dietary patterns with weight control behaviors including physical activity. It has been demonstrated that people with healthy dietary patterns (consuming more vegetables and fruits) are more likely to conduct the physical activity.^14^ In a study, a reverse correlation was found between following the western dietary patterns and a low level of physical activity while there was not a significant relationship between a mixed dietary pattern and the level of physical activity.^15^

Considering the associations between body weight, nutrition, and risk of bone fracture among postmenopausal women with osteoporosis, weight control, and dietary patterns could be imperative in reducing the risk of bone fracture.^16^ However, literature has shown limited effectiveness of common approaches on long-term overweight and obesity management, which highlights the difficulty in effective weight control.^16-18^ The difficulty in obtaining weight in a normal range may be due to the fact that weight control behaviors, like nutritional habits and physical activity, are influenced by a wide range of socio-economical, personal, environmental, and psychological factors.^19^ So, the application of theoretical frameworks may be useful in systematic investigation of the issue and its determinants in an organized manner, as suggested by Glanz et al.^20^ A wide range of theoretical frameworks have been applied to examine different health behavior issues,^21-24^ within which the theory of planned behavior (TPB) is a well-known approach.^25-27^

Similar to many health-related behaviors, weight control could arise from the individuals’ intention and the antecedents of intention, including personal attitude, subjective norms (SNs), and the ultimate perceived behavioral control (PBC). This is called TPB, which is based on the assumption that human behavior results from the human intention to undertake the behavior and his/her ability to do it, consciously.^16^ Based on this theory, the intention is influenced by three factors: attitude, and PBC.^28^ The intention is simply defined as how hard the people are willing to plan for changing or adapting to a behavior.^29^ Attitude is one’s positive or negative evaluation of behavior. SN is “the perceived social pressure to perform the behavior or not”,^28^ and PBC refers to people’s perceptions of their ability to perform a given behavior.^30^ In this study, we aim to assess the cognitive determinants of weight control behaviors and to determine relationships between dietary patterns and weight control behavior among postmenopausal women with osteoporosis.

## Materials and Methods

### Participants and procedures

This cross-sectional study was performed in Tabriz, Iran, from July to December 2017. According to the prevalence of osteoporosis in a previous study (34.1%),^31^ confident intervals (CIs) = 95%, and standard error = 6%, the sample size was determined to be 270. Through a convenience sampling method, 270 postmenopausal women with osteoporosis in two bone densitometry centers in the city were recruited to participate in the study. In the rheumatology clinic, women were requested to perform bone densitometry if they had at least one of the International Society for Clinical Densitometry (ISCD) indications.^32^ Among 270 invited patients, 21 cases declined to participate in the study, 9 cases did not meet inclusion criteria and were thus excluded, leaving a total of 240 study participants (response rate = 92.2%).

In women aged 40 to 45 years, the eligibility criteria to participate in the study were being menopaused for at least six months, based on FSH (Follicle-simulating hormone) test approved by a gynecology and obstetrician specialist. In women over 45, the criteria were having no menstrual period for at least 12 months and not using hormonal contraception, or having had a menopause diagnosis based on symptoms in those without a uterus. All diagnoses were conducted by a gynecology and obstetrician specialist. Those with primary ovarian insufficiency, diagnosed by the specialist, did not include in the study. The BMD measurements of the left proximal femur (the femoral neck, or FN), the lumbar spine (LS; L2–4), and total hip were performed using dual-energy X-ray absorptiometry (Hologic QDR 2000; Hologic, Waltham, MA, USA). Instruments were calibrated daily and had measurement precisions of 0.008 g/cm^2^ for the spine and 0.013 g/cm^2^ for the femoral neck. Eligibility criteria were having a T-score ≤ -2.5 at the mean lumbar spine (L1-4), femoral neck, or total, according to the definition of the WHO as diagnosed by a rheumatologist. Exclusion criteria included being surgically menopause, taking antidepressants and psychotropic drugs, immunosuppressive agents and corticosteroids, being with type 2 diabetes mellitus, rheumatoid arthritis, history of rheumatism and/or lupus, ankylosing spondylitis, and spondylitis arthritis, and being under special diets. All participants provided written informed consent before enrollment in the study.

### Biometric characteristics

Demographic data were collected by a health care expert. Demographic data included age, t-score, z-score, age at menopause, marital status, education status, BMI (kg/m^2^), supplement therapy, occupation, and BMD at the spine (L1-4), femoral neck, or total.

### Weight-CuRB Questionnaire

To assess weight control behavior and its determinants among postmenopausal women with osteoporosis, a previously validated and reliable TPB-based instrument, namely Weight-CuRB,^24^ was applied. This questionnaire comprised five subscales (19 items) including attitude (5 items), intention (4 items), SN (3 items), PBC (3 items), and weight control behaviors (3 items). A ﬁve-point Likert-type scaling (from completely disagree [0] to completely agree [4]) was the response format for the items of attitude, intention, and SN scales. The higher the scores the more positive attitude and the higher levels of intention and SN were implied toward weight control behaviors. The response formats for PBC (from totally incorrect [0] to totally correct [4]) and weight control behaviors (from never [0] to always [4]) were also based on a ﬁve-point Likert scale, where the higher scores indicated the higher levels of PBC and performing weight-control behaviors among the patients.

### Nutrition intake assessment

A 40-item food frequency questionnaire (FFQ) was used to determine the common dietary patterns of postmenopausal women with osteoporosis. The questionnaire was filled in for the patients by a health care expert. The FFQ aimed to assess the food intake within a year, so the participants were asked to mention the intake frequency of each item daily, monthly, or yearly.

### Statistical analysis

For dietary pattern analysis, food items consumed by participants were classified into twenty-two food groups.^33^ To identify dietary patterns, we entered the data on the food groups^34^ into the exploratory factor analysis (EFA) utilizing a principal component analysis with varimax rotation. We estimated the score of patterns with a weighted method, applying the weight of factor loadings related to each item. Among the others, the pattern with a high score was considered the dominant dietary pattern.^34^ Pearson correlation coefficient test was used to assess the relationships between the TPB-based variables and weight control behavior. Multiple linear regression analysis was performed to investigate the predictors of weight control behaviors based on the TPB variables by dietary pattern. In this analysis, weight control behavior was considered as the dependent variable. Attitude, SN, PBC, and intention were entered as independent variables. The best-fit model explaining the relationships among the variable was achieved using enter strategy from the variables. Also, to investigate the correlates of intention by dietary patterns, the intention was considered as the dependent variable and all other TPB-based variables (include SN, attitude, and PBC) were entered as independent factors. To test for multicollinearity, we used the collinearity diagnostics test in the regression analysis which represented the variance inflation factor (VIF). The VIF factors were between 1 to 4 in both the tests for (a) intention to control weight and (b) weight control behaviors. Although some multicollinearities were found, the factors did not exceed 10, so we decided to accept them for our further analysis. The level of significance was considered at 0.05, a priori. All data analyses were conducted using IBM SPSS Statistics 20 (SPSS Inc., Chicago, IL, USA) for windows.

## Results

### Participants

One hundred and twenty of the participants were with BMI ≥25; 99 had a family history of osteoporosis; 140 were under supplement therapy with Ca and vitamin D (Table 1). The mean (standard deviation; SD) age, t-score, z-score, age at menopause, and BMD at the spine (L1-4), femoral neck, or total were 60.13 (6.57), -2.96 (0.50), -1.22 (0.77), 46.76 (5.73) and 0.56 (0.16), respectively.

**Table 1:** Summary of demographic characteristics of the study population (n = 240)

**Variables**		**N**	**%**
Age (years)	≤45	61	25.4
	46-55	59	24.6
	56-65	67	27.9
	66+	53	22.1
Body mass index (kg/m^2^)	<18.5	61	25.4
	18.5-24.9	59	24.6
	25-29.9	60	25
	30+	60	25
Marital status	Single	5	2.1
	Married	204	85
	Widow	31	12.9
Education status	Illiterate/elementary	146	60.8
	Diploma	53	22.1
	University education	41	17.1
Occupation	Housewife	162	67.5
	Worker/farmer/self-employment	14	5.9
	Employee	15	6.3
	Retired	49	20.4
Supplement therapy	Ca + Vitamin D	140	58.3
	Vitamin D	57	23.8
	Vitamin B and others	28	18.7
Age at menopause (years)	≤40	30	12.5
	41-50	187	77.9
	≥51	23	9.6

### Dietary patterns

The results of EFA indicated three dietary patterns. The first pattern included liquid vegetable oil, fruits, nuts, low- and high-fat dairy, olives and olive oil, and unrefined grains. This pattern was named a ‘healthy pattern’. The second pattern, named as ‘mixed pattern’ included red meat, legumes, refined grains, fish and hen meat, solid oil, and vegetables. The third pattern, named as ‘western pattern’ included sweets and desserts, produced meat, tea and coffee, cans, potato, and soft drinks.^34^ In total, 24.44% of the variance was explained by the patterns, within which 8.92%, 7.73%, and 7.59% were explained by western, healthy, and mixed dietary patterns, respectively (Table 2).

**Table 2:** Factor loading coefficients to determine dietary patterns in postmenopausal with osteoporosis (n = 240)

**Items**	**Dietary patterns**		
	**Mixed**	**Healthy**	**Western**
Egg	0.577	0.210	-0.328
Red meat	0.519	-0.384	0.151
refined grains	0.494	-0.265	0.202
Vegetables	0.399		
Mayonnaise	-0.383		0.302
Legumes	0.377	0.169	
Fish & hen meat	0.377		-0.210
Fast foods	-0.257	-0.108	
Solid fats	0.236	-0.219	-0.172
Unprocessed grains	-0.281	0.660	-0.107
Nuts		0.527	
liquid vegetable oil	0.258	0.374	
Fruits		0.306	
Olive and olive oil		0.285	-0.107
high-fat and low-fat dairy	0.260	0.265	
Pickles and salt		-0.162	
Soda			0.670
Potato	0.379	-0.113	0.606
canned food	-0.219	-0.166	0.408
Organ meats	-0.107	-0.219	0.382
Tea & coffee			0.170
Sweets and desserts		-0.124	0.155
**N (%)**	78(32.7)	71(29.5)	91(37.8)
**Variance in food intake**	7.594%	7.735%	8.917%
**Total variance predicted**	24.24%		

### Predictors of weight control behaviors by dietary patterns

PBC had significant correlations with attitude (r = 0.202, *P* < 0.01) and intention (r = 182, *P* < 0.01). Attitude had also significant correlation with SN (r = 0.136, *P* < 0.05). Table 3, the first part, indicates the TPB-based determinants of weight control behavior by dietary patterns. In the healthy and western dietary patterns, attitude (β: 0.140, *P* < 0.001) and SN (β: 0.498, *P* < 0.01) were the only predictors of weight control behavior, respectively. Moreover, in the women with healthy and western patterns, the TPB-based variables altogether explained 11% and 16% of variations in weight control behaviors, respectively.

**Table 3:** TPB-based determinants of weight control behavior and intention to control weight by dietary patterns in postmenopausal women with osteoporosis (n = 240)

**Dominant dietary patterns**	**TPB-subscale**	**β**	**Standard error**	**Beta**	***P*** ** value**	**R**	**R** ^ 2 ^
**Dependent variable: Weight control behavior**							
Healthy diet (n = 71)	Attitude	0.316	0.046	0.140	0.001	0.175	0.111
	Intention	0.046	0.116	0.051	0.696		
	Subjective norms	0.099	0.158	-0.079	0.534		
	Perceived behavioral control	0.076	0.081	0.119	0.351		
Mixed diet (n = 78)	Attitude	0.059	0.069	0.144	0.396	0.201	0.041
	Intention	0.053	0.118	0.061	0.658		
	Subjective Norms	0.011	0.156	0.011	0.946		
	Perceived behavioral control	0.105	0.089	0.167	0.242		
Western diet (n = 91)	Attitude	0.080	0.065	0.228	0.222	0.404	0.163
	Intention	0.166	0.124	0.188	0.188		
	Subjective norms	0.417	0.151	0.498	0.008		
	Perceived behavioral control	0.082	0.091	-0.120	0.376		
**Dependent variable: Intention to control weight**							
Healthy diet (n = 71)	Attitude	0.140	0.063	0.262	0.059	0.388	0.150
	Subjective norms	0.173	0.082	0.242	0.038		
	Perceived behavioral control	0.201	0.162	0.143	0.220		
Mixed diet (n = 78)	Attitude	0.158	0.075	0.330	0.040	0.281	0.079
	Subjective norms	0.038	0.100	-0.052	0.707		
	Perceived behavioral control	0.106	0.176	0.090	0.549		
Western diet (n = 91)	Attitude	0.183	0.069	0.462	0.071	0.371	0.138
	Subjective Norms	0.094	0.103	-0.122	0.367		
	Perceived behavioral control	0.358	0.164	0.377	0.034		

Abbreviation: TPB: Theory of planned behavior.

### Predictors of intention to control weight by dietary patterns

In the healthy and western dietary patterns, SN (β: 0.242, *P* < 0.05) and PBC (β: 0.377, *P* < 0.05) were the only predictors of intention to control weight (Table 3). In the women with healthy and western patterns, all TPB-based variables explained 15% and 13.8% of variations in intention to control weight.

### Predictors of weight control behavior

Stepwise regression of weight control behavior, as the dependent variable, on the TPB constructs (attitudes, SN, intention, and PBC), as independent variables, showed that the TPB-based variables explained 12.2% of variations in weight control behavior (Table 4, the first part), within which SN was the only significant predictor of the behavior (β = -0.199, *P* <0.01).

**Table 4:** Multiple linear regression analysis to determine the predictors of weight control behavior and intention to control weight in postmenopausal women with osteoporosis (N = 240)

**TPB-subscales**	**β**	**Standard error**	**Beta**	***P*** ** value**	**R**	**R** ^ 2 ^
**Dependent variable: Weight control behavior**							
Attitude	0.005	0.039	0.018	0.906	0.188	0.122
Intention	0.099	0.063	0.177	0.177		
Subjective norms	-0.105	0.035	-0.199	0.003		
Perceived behavioral control	0.144	0.085	0.133	0.091		
**Dependent variable: Intention to control weight**							
Attitude	0.172	0.039	0.272	0.000	0.321	0.131
percieve behavior control	-0.074	0.036	-0.130	0.038		
Subjective norms	0.210	0.086	0.152	0.016		

TPB: Theory of Planned Behavior

### Predictors of intention

Stepwise regression of intention to control weight, as the dependent variable, on the TPB constructs (attitudes, SN, and PBC), as independent variables, indicated that the three variables explained 13.1% of variations in intention to control weight, and all three variables were significant predictors of the dependent variable (Table 4).

## Discussion

This study investigates the cognitive factors of weight control behavior by dietary pattern among postmenopausal women with osteoporosis. Healthy (32.7%), mixed (29.5%), and western (37.8%) dietary patterns were identified among participants. In the women with healthy (% of variance explained = 11) and western (% of variance explained = 16) dietary patterns, attitudes, and SN were found to be associated with weight control behavior, respectively. Moreover, in the women with healthy (% of variance explained = 15) and western (% of variance explained = 13.8) dietary patterns, SN, and PBC were related with intention to control weight, respectively. As our results show, SN seems to play a remarkable role in determining weight control behaviors. It means that the patients with a western unhealthy dietary pattern are more influenced by their surroundings and significant others, and thus are less likely to perform weight control behaviors. On the other hand, among patients with a healthy dietary pattern, attitude alone was in association with the behavior, which means that, compared to those with a negative attitude, the patients with a positive attitude towards weight control are more likely to demonstrate the behaviors. Fila and Smith assessed the correlates of healthy eating behaviors and showed that attitude and SN were associated with eating behaviors among women.^35^ A novel finding that we can add to the literature is that the patients with a healthy dietary pattern are more reliant on their own attitude and perceptions to control their weight, compared to the patients with a western dietary pattern who are mainly decide based on the social norms and pressures surrounding them. This finding is imperative for the researchers, health policymakers, and health practitioners interested in planning weight control interventions among women with osteoporosis.

Moving further, among all participants, the TPB-based factors altogether explained about 12% and 13% of weight control behavior and intention to control weight, respectively. SN was the only significant predictor of the behavior (add relevant statistics here). For the intention to control weight, however, all three factors were significant determinants. While evaluating changes in health-related behaviors, Schifter and Ajzen^36^ noted that PBC and intentions were associated with the behavior of low-fat diet and that intentions were associated with attitudes. Such differences in the cognitive determinants of health-related behaviors may be due to the differences in the primary outcomes and populations of the studies. Palmeira et al^37^ reported 14.8% of the variance in weight controlling behavior by SN, intention, PBC, and attitude, other previous studies also reported heterogeneous results. Unlike our findings, intention and PBC were observed as two important determinants of weight reduction.^36,37^ However, Psouni et al in a study to assess exercises and healthy eating behaviors and their related intentions among normal weight and overweight/obese adults found that in the overweight/obese group, SNs had indirect relation with exercise behavior, whereas PBC was directly related to exercise behavior. Similar to our findings, attitude, SN, and PBC were the correlates of intention to exercise in both the overweight/obese and normal-weight groups.^38^ However, a highlight finding in our study was the remarkable association of SN in predicting both, weight control and intention to perform it. SN is associated with the belief that significant others or groups of people either approve or disapprove of a particular behavior.^39^ So, it seems that the women who participated in our study have perceived social pressure on weight control from significant others, which may motivate them to intend for complying with the behavior. These findings are supported by several previous studies conducted to determine the predictors of healthy behaviors.^40-42^

McConnon et al^16^ also showed expectation as the best predictor of weight control and PBC as the most consistent associate of expectation.^16^ Despite these dissimilarities, our results are in line with some of those reported by McConnon et al who found that PBC, attitude, and SN were significantly related to the intention, expectation, and desire in post-weight maintenance. They also observed that SN was in a negative association with weight regain. Similarly, in our study, an indirect relationship was found between SN and weight control behavior, suggesting that as SN increases, weight control behavior decreases. In our results, SN had also a positive relationship with the intention to control weight. McConnon et al^16^ also found associations between SN and intention to post-weight maintenance. All these findings suggest that as SN increases, weight control intention and weight maintenance increase. Healthcare providers and health practitioners should consider the significant role of SN while designing weight control interventions among postmenopausal women with osteoporosis.

The western pattern was the most common dietary pattern, followed by healthy and mixed patterns among Iranian women with osteoporosis. It is believed that a wide range of bio-behavioral factors, including the dietary patterns people, adopt,^43^ physical activity, and lifestyle,^44^ as well as biological factors, could be associated with the prevalence rates of hip bone fractures, which imposes a high level of a financial burden on societies.^45^ In the present study, almost two-thirds of Iranian women followed western and mixed dietary patterns. One of the most important factors in forming dietary patterns within societies is a collection of nutritional habits and culture and nutritional literacy.^46^ In a previous study, the authors reported similar findings and showed that the diet trends have switched from healthy to western patterns. They also associated this nutritional transition with rapid population changes, urbanization, and improvements in the social status of populations, which all have made big changes in the food basket of families- with a high level of energy sources and a low level of fruits and vegetables.^47^

In the present study, no relationship was found between intention and weight control behaviors. Similarly, Lien et al^48^ found no association between intention and vegetable/fruit consumption among young adults. Despite high intention, because of poor perception, individuals may not expect the desired behavior. It is assumed that food intake is rooted in people’s habits, but the intention is affected by resources of personal and environmental control. Since weight control is a non-volitional behavior, it is difficult to identify a direct relationship between intention and behavior.^49^ To the best of the authors’ knowledge, this is the first study of its kind within which the cognitive correlates of weight control behaviors by the dietary pattern are investigated among women with osteoporosis in a developing country. Due to the nature of the study, as cross-sectional research, inferring causality is warranted. Furthermore, many participants were from rural areas, so they could not recall food intake and the medications they consume daily.

## Conclusion

Our findings highlighted the remarkable role of dietary patterns in the associations between weight control and its cognitive determinants among postmenopausal women with osteoporosis. As weight control behaviors and healthy food patterns can reduce the risk of bone fractures among osteoporotic women,^50,51^ it is imperative to consider dietary patterns while designing weight control educational interventions. While designing such interventions, promoting SN and PBC should be considered as the core strategies to promote the behaviors among the patients who follow an unhealthy diet. Among those with a healthy dietary pattern, attitude and SN are recommended as the core categories of the intervention.

## Acknowledgements

The authors would like to thank all patients who participated in this study.

## Funding

This research did not receive any specific grant from funding agencies in the public, commercial, or not-for-profit sectors.

## Competing interests

Haidar Nadrian is an Associate Editor in Health Promotion Perspectives. The other authors declare that there is no conflict of interest.

## Ethical approval

This study was approved by Ethics Committee in Tabriz University of Medical Science; Approval ID: IR.TBZMED.REC.1398.920. At the outset, written informed consent was obtained from all participants.

## Authors’ Contributions

HH: methodology, data acquisition, analysis, writing – original draft, writing – review & editing, project administration. HN and SK: methodology, writing – review & editing, supervision. FSB and KG: data acquisition, writing – review & editing. PSA: methodology, analysis, data, writing, review & editing, supervision. All authors gave final approval to the version to be published, and agree to be accountable for all aspects of the work.

## Disclaimer

The authors claim that no part of this paper is copied from other sources.
